# Supplementary material for: The interactome of intact mitochondria by cross-linking mass spectrometry provides evidence for coexisting respiratory supercomplexes
Source: Mol Cell Proteomics. 2017 Dec 8;17(2):216–32. doi: 10.1074/mcp.RA117.000470 (PMC5795388; doi:10.1074/mcp.RA117.000470)
Supplement: Supplemental Data [file supp_RA117.000470_133922_0_supp_23978_fzffwf.zip › spectra_annotation/mito_spectra_annotation/170-1-4-1-22-1.pdf]

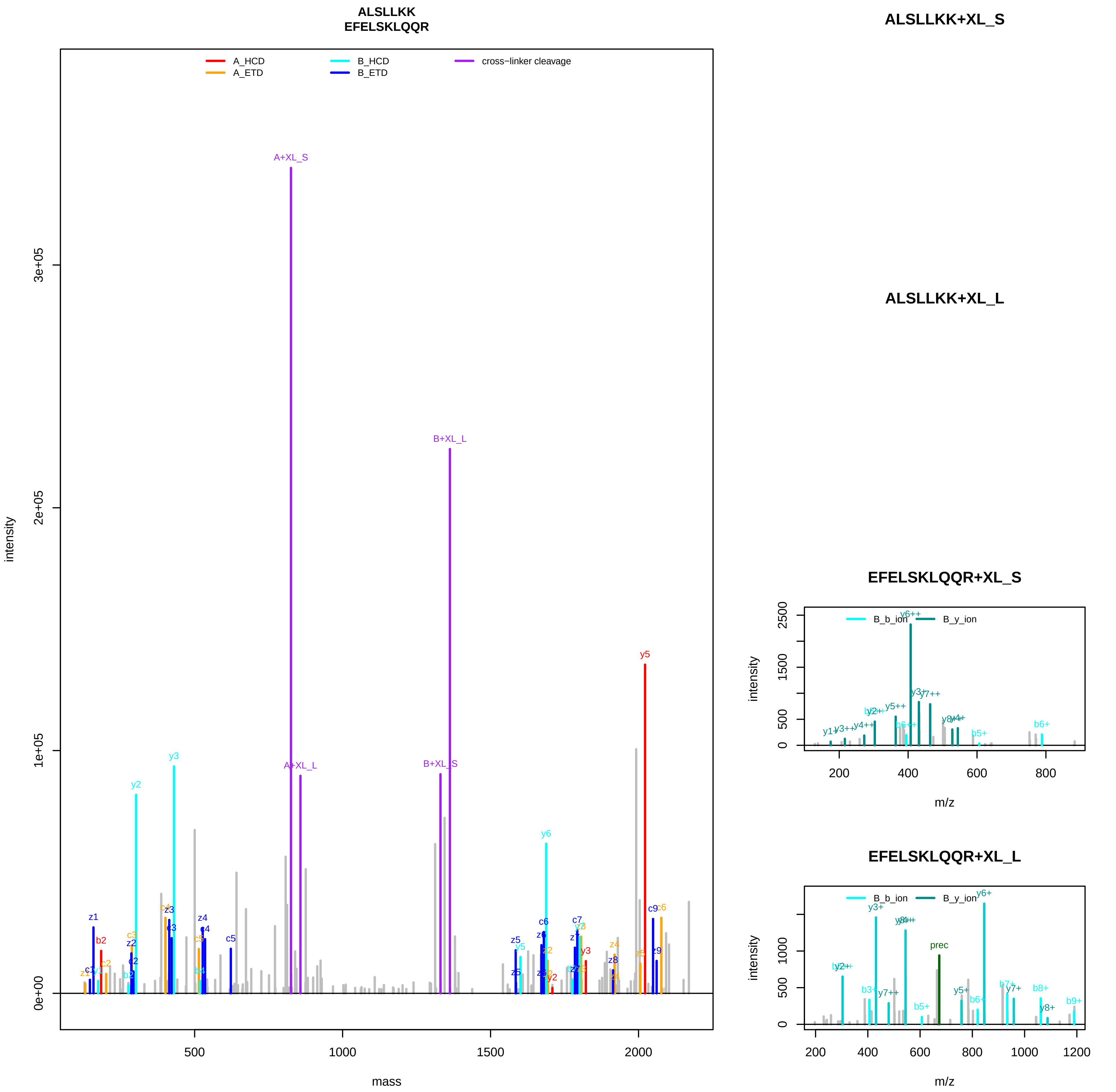
ALSLLKK
EFELSKLQQR
A_HCD
A_ETD
B_HCD
B_ETD
cross−linker cleavage
A+XL_S
B+XL_L
A+XL_L
B+XL_S
y5
y6
y3
y2
intensity
mass
0e+00
1e+05
2e+05
3e+05
500
1000
1500
2000
ALSLLKK+XL_S
ALSLLKK+XL_L
EFELSKLQQR+XL_S
B_b_ion
B_y_ion
y6++
intensity
m/z
0
500
1500
2500
200
400
600
800
EFELSKLQQR+XL_L
B_b_ion
B_y_ion
y6+
y3+
prec
intensity
m/z
0
500
1000
200
400
600
800
1000
1200
